# Leber’s hereditary optic neuropathy: Update on current diagnosis and treatment

**DOI:** 10.3389/fopht.2022.1077395

**Published:** 2023-01-11

**Authors:** Ali Esmaeil, Ali Ali, Raed Behbehani

**Affiliations:** Neuro-Ophthalmology Service, Department of Ophthalmology, Ibn Sina Hospital, Kuwait City, Kuwait

**Keywords:** idebenone, genetic vector therapy, mitochondrial disorder, diagnostics, Leber’s hereditary optic neuropathy

## Abstract

Leber’s hereditary optic neuropathy (LHON) is a fairly prevalent mitochondrial disorder (1:50,000) arising from the dysfunction of the mitochondrial respiratory chain, which eventually leads to apoptosis of retinal ganglion cells. The usual presentation is that of a young male with a sequential reduction in visual acuity. OCT has been used to study the pattern of optic nerve involvement in LHON, showing early thickening of the inferior and superior retinal nerve fibre layer and ganglion cell layer thinning corresponding with the onset of symptoms. Of the three primary mutations for LHON, the m.14484T>C mutation has the best visual prognosis. Recent emerging therapeutic options for LHON include idebenone and the introduction of genetic vector therapy, which is currently in phase III clinical trials. Screening of family members and adequate advice to avoid environmental triggers, such as smoking and alcohol consumption, are also cornerstones in the management of LHON.

## Introduction

Leber’s hereditary optic neuropathy (LHON) is a maternally inherited mitochondrial disorder that manifests as subacute, sequential, and painless bilateral vision loss, typically in young males (1, 2). Von Graefe initially recognized the condition in 1858; however, it was later named after Dr. Theodore Leber (3), who reported the condition in several patients across different families and described its unique clinical characteristics (4). LHON was initially thought to be an x-linked disorder, but with later understanding of mitochondrial inheritance, it became clear that mitochondrial mutations are the underlying cause (5).

LHON is one of the most prevalent mitochondrial disorders in specific populations (6, 7). We review the recent developments in the understanding of the pathophysiology of LHON and the latest updates in the diagnostic and therapeutic strategies of LHON.

## Epidemiology

The worldwide prevalence of LHON is estimated at 1 in 50,000, with some variability across different countries and continents. In a molecular genetic epidemiological study of LHON in the UK, the prevalence was 1 in 31,000 (6). Another study carried out in Finland suggests the local prevalence to be closer to 1 in 50,000 (8). In Denmark, the prevalence of LHON was reported as 1 in 54,000 (9). 1 in 68,000 was the prevalence of LHON reported in Australia (10). A recent nationwide questionnaire survey carried out in Japan estimates the prevalence LHON to be 1:50,000 (11).

LHON has been widely regarded as a disease of young males peaking at the age of 14-26 years, with a male-to-female ratio of 5:1. However, recent studies have shown that the ratio is closer to 3:1, and approximates 1:1 after the 3^rd^ decade of life (12). Moreover, although the disease is more prevalent in young adults, it can manifest at any age, and 10% of disease onset occurs after the age of 50 (12).

## Genetics

Mitochondrial DNA (mtDNA) is a double-stranded circular molecule with a genome containing 37 genes (13). Oxidative phosphorylation is a process mediated by the enzyme complexes (I–V) on the inner mitochondrial membrane. Subunits of complex II are encoded entirely by nuclear DNA, while complexes I, III, IV, and V are encoded by a combination of nuclear and mtDNA. Complex I, the site of all the primary LHON mutations, is a multimer of 7 mitochondrial-encoded subunits and a minimum of 36 nuclear-encoded subunits (14).

Mitochondrial mutations in LHON were first discovered by Wallace et al. in 1988 (5), and are therefore inherited strictly through a maternal lineage. The three primary point mutations in the mitochondrial genome (m.11778G>A, m.14484T>C, m.3460G>A) constitute 95% of all LHON mutations (15). The m.11778G>A mutation, which involves the MT-ND4 gene, accounts for approximately 70% of LHON cases and has the worst prognosis for visual recovery (15). The m.14484T>C, which affects the MT-ND6 gene, is responsible for 14% of LHON cases and has the best prognosis for visual recovery (16–18). Finally, the least prevalent primary LHON mtDNA mutation is the m.3460G>A (13% of cases), which involves the MT-ND1 gene. The rate of partial visual recovery (0.3 LogMAR change in visual acuity) in m.11778G>A, m.14484T>C, and m.3460G>A, is 4-25%, 37-58%, and 20%, respectively (6, 19–21).

In the majority of LHON pedigrees, the primary mutation responsible for LHON is homoplasmic (mutation is present on all inherited mtDNA) (19); however, heteroplasmy (mutation is present in a fraction of the mitochondria DNA) is found in about 10-15% of LHON cases (22). The mutation load appears to be correlated with penetrance and the phenotypic expression of the disease and with the risk of disease manifestation significantly reduced if the mutational load is less than 60% (23). However, LHON with heteroplasmic inheritance does not necessarily manifest as a milder form of the disease (24).

The incomplete penetrance of LHON is not well understood, but some factors affect the expression of LHON mutations. The association of LHON with certain haplogroups (haplogroup J) might have a role in modifying the risk of phenotypic expression of the disease. Mitochondrial haplogroups can be defined as a group of similar haplotypes (a group of alleles inherited in combination from a single parent) with single nuclear polymorphisms inherited from a common ancestor. Sequential accumulation of mutations through maternal lineages is responsible for the development of these haplogroups (25).

Haplogroup J, which is associated with the mutations m.4216T>C, m.13708G>A, m.15257G>A, and m.15812G>A, has been classically thought to enhance the penetrance of m.11778G>A and to a lesser extent m.14484T>C (26). However, this has been challenged by the finding that the presence of haplogroup J with other primary mutations does not seem to further impair mitochondrial oxidative metabolism, nor influence the age of onset or the final visual outcome of LHON (26). Finally, less common secondary mutations (m.11696G>A, m.14502T>C, m.3497C>T, m.3394T>C, m.12811T>C, m.11696 G>A, and m.3316G>A) have been associated with LHON, and they are postulated to act in synergy with the three primary mutations responsible for the disease (27, 28).

Gender has been recognized as an important modifier for the risk of penetrance of LHON. Approximately 10% of females and 50% of males with an underlying LHON mutation will experience vision loss (15). The gender predilection has been linked to the protective effects of estrogen *via* the activation of mitochondrial biogenesis and increasing the mitochondrial load, decreasing the production of reactive oxygen species, and reducing apoptosis in retinal ganglion cells (RGC) (29). In addition, x-linked modifier genes, such as PRICKLE3, encode for mitochondrial proteins linked to the biogenesis of ATPase. In experimental animal modes, PRICKLE3-deficient mutants had a greater rate of conversion to LHON (30).

Environmental risk factors associated with an increase in LHON penetrance include smoking, heavy alcohol consumption, chemical toxins, as well as antiretroviral and antituberculosis medication (31, 32). Cigarette smoking, in particular, is the most established risk factor, and it has been shown to reduce the mtDNA load in the blood cells of LHON patients as well as reduce the mtDNA load and ATP levels in fibroblast models of LHON patients (33). Smoking also has a deleterious effect on the bioenergetic compensation of LHON carriers, and in animal models, cigarette smoking did not reduce ATP levels in non-mutant control fibroblasts (33).

## Pathophysiology

Polypeptide complexes I-V are situated in the inner mitochondrial membrane and are responsible for ATP production through the process of oxidative phosphorylation. In the respiratory chain, electron donors such as NADH and FADH_2_ contribute electrons to complexes I and II, respectively. Shuttling of electrons through the rest of the chain is aided by co-enzyme Q_10_ and cytochrome *c*. The energy produced by electron shuttling allows protons to be pumped from the mitochondrial matrix to the intermembrane space. The final step in the oxidative chain involves the utilisation of the electrochemical proton gradient by complex V (ATPase) to catalyse the conversion of adenosine diphosphate (ADP) into adenosine triphosphate (ATP) (34).

**Figure 1:**
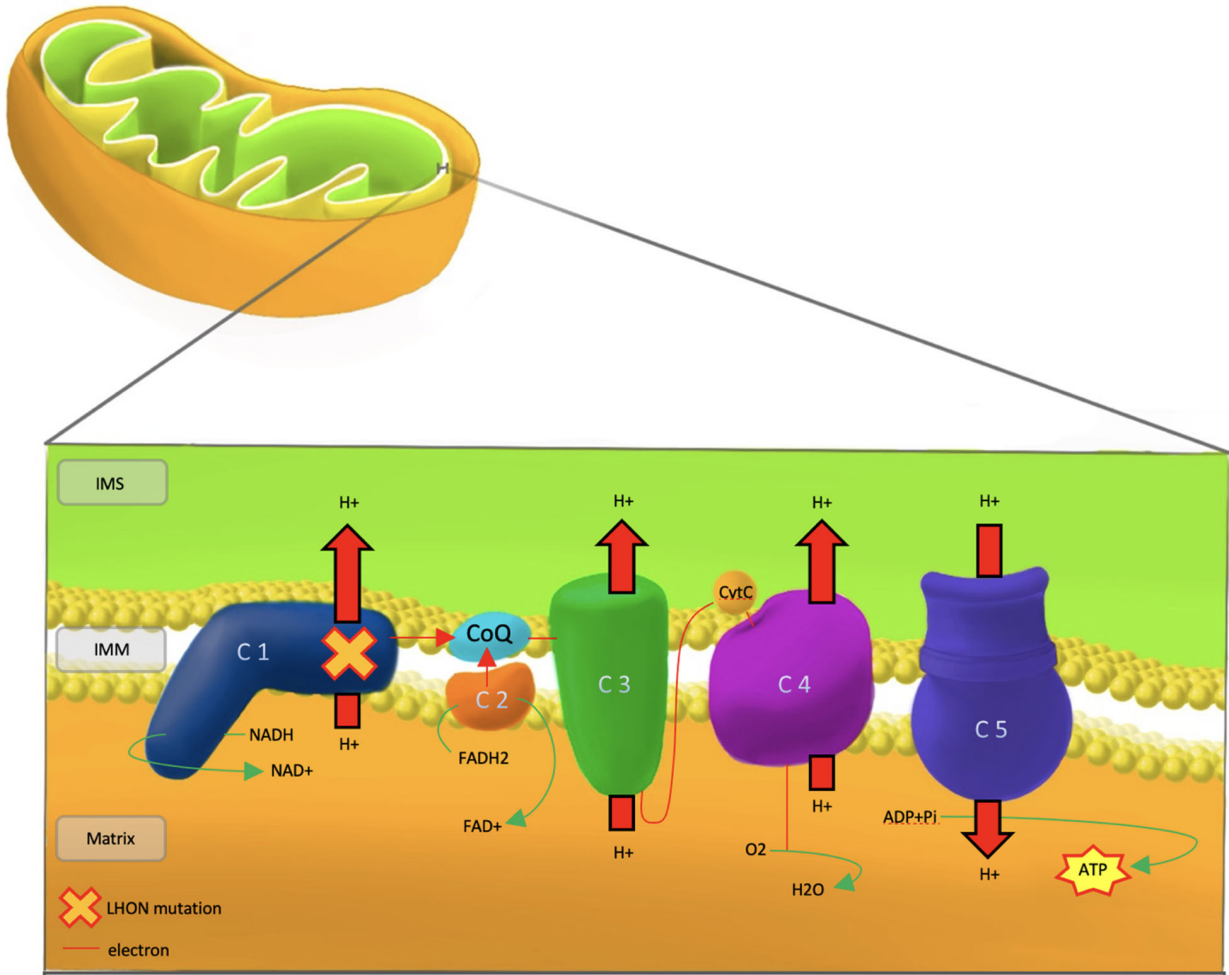
Pathophysiology of LHON, showing the mutational defect in Complex 1 of the respiratory chain. IMS, Inter Membrane Space; IMM, Inner Mitochondrial Membrane; LHON, Lebers Hereditary Optic Neuropathy; C 1-5, Complex 1-5; CoQ, co-enzyme Q10; CytC, cytochrome c; NADH/NAD+, Nicotinamide adenine dinucleotide; FADH2/FAD+, flavin adenine dinucleotide; ADP, Adenosine Diphosphate; ATP, Adenosine Triphosphate; Pi, Phosphate; H+, Hydrogen; O2, Oxygen; H20, water.

Dysfunction of the respiratory chain caused by LHON mutations of complex I subunits leads to defects in energy production and downstream accumulation of reactive oxygen species (ROS) that ultimately leads to RGC apoptosis (**Figure 1**).

Under normal intracellular conditions, the presence of antioxidant enzymes, such as superoxide dismutase (SOD) and catalase (CAT), counteract the build-up of ROS. In LHON, net build-up of free electrons from poor electron shuttling and the resultant generation of large amounts of ROS leads to RGC death, even without a significant reduction in ATP production (35). Additionally, reduced SOD activity was found in animal models exhibiting optic neuropathy with a pattern similar to that of LHON, which emphasizes the importance of ROS in the pathophysiology of the LHON (36). The build-up of ROS causes damage to various intracellular membranes and the release of calcium from intracellular stores. The rise of intracellular calcium contributes to mitochondrial permeability transition pore opening and the release of intrinsic apoptotic triggers such as cytochrome c (37). Furthermore, low energy production arising from dysfunctional oxidative phosphorylation leads to a net influx of axonal calcium due to the altered function of membrane channels, namely the Na+-Ca2+ exchanger (38).

The predilection of LHON to involve the papillomacular bundle fibers of the optic nerve axons suggests that the diameter of the axons and myelination play a role in the pathophysiology. The unmyelinated axons of RGCs exist in the pre-laminar segment and are myelinated in the post-laminar segment (39). The lack of myelin in the pre-laminar segment renders them less efficient in propagating action potential and, therefore, especially vulnerable to damage in LHON due to the higher energy needs (31).

RGCs are classified into midget, parasol, and small bistratified ganglion cells. Midget RGCs carry the smallest calibre axons and are the primary type of RGCs affected in LHON. In addition, midget cells are the most prominent subtype in the papillomacular bundle mediating visual information and red-green chromaticity (40). In the early pre-symptomatic phase of the disease, temporal macular RGCs and peripapillary nerve fibres are initially damaged.

## Natural history, examination, and diagnostic evaluation

LHON classically presents with bilateral sequential vision loss, with the interval between the two eyes varying from weeks up to years apart. Visual acuity is often severely affected due to the early involvement of the papillomacular bundle, which results in a dense central or centro-cecal scotoma that enlarges over time (41, 42). The pupillary light reflex is often brisk and preserved, with the absence of a relative afferent pupillary defect, even with early unilateral involvement. This has been attributed to the preservation of melanopsin-containing RGCs early in the clinical course of LHON (43). Melanopsin RGCs constitute one percent of all the RGCs and are sensitive to sustained and strong blue light, contributing to autonomic functions like the circadian rhythm and pupillary constriction (44).

The natural course of LHON generally follows a pre-symptomatic phase, an acute phase, and a chronic phase. The duration of each phase can vary from patient to patient, but a general timeline is 12-24 weeks for the acute phase and a transition to the chronic phase after the initial 6 months (45). The acute phase is characterised by the deterioration of central vision and is usually when patients first present.

The clinical findings in a patient with LHON in the pre-symptomatic phase include peri-papillary telangiectatic vessels (peripapillary microangiography) and mild zones of disc pseudo-oedema (retinal nerve fibre layer swelling around the optic disc without leakage of fluorescein angiography) (Sadun et al., 2004). Spectral-domain OCT at this stage will demonstrate dynamic inferior-temporal retinal nerve fibre layer (RNFL) thickening and no significant changes in the ganglion cell layer (GCL) (46). Swelling of the RNFL may reflect a compensatory aggregation of mitochondria in the nerve fibres. This may be attributed to enhanced mitochondrial biogenesis, which is activated as a compensatory strategy to mitochondrial dysfunction in LHON (47).

**Figure 2:**
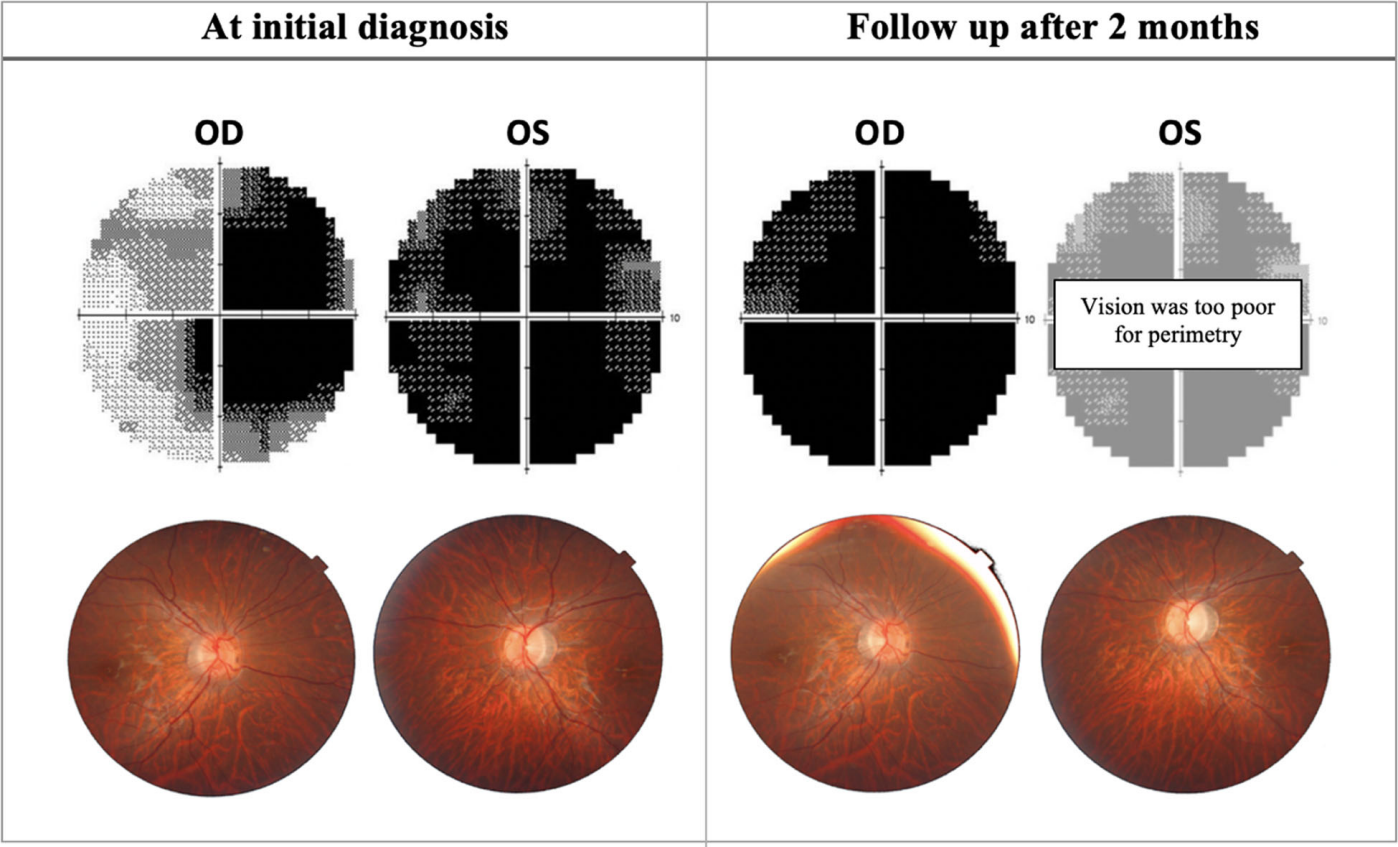
Progression of perimetric and fundoscopic findings in patient A, an 18-year-old LHON patient. The patient presented with poor visual acuity at initial diagnosis: 20/400 OD & CF OS. Right eye later progressed to CF after 2 months. *OD, Oculus dexter; OS, Oculus sinister; CF, Counting fingers*.

**Figure 3:**
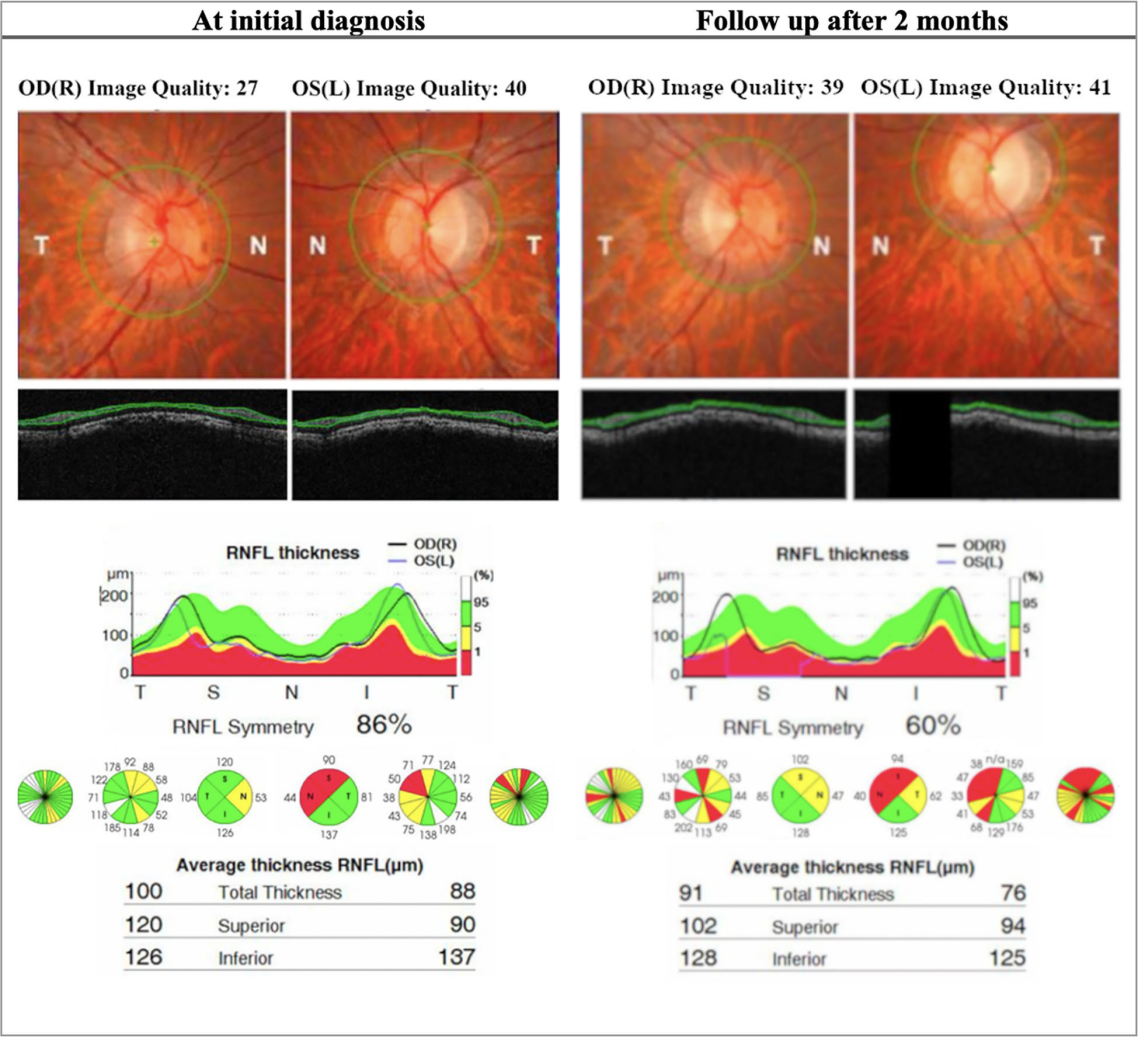
OCT (Topcon 3DOCT-3000) findings in patient A, an 18-year-old LHON patient. The patient presented with poor visual acuity at initial diagnosis: 20/400 OD & CF OS. The right eye later progressed to CF after 2 months. This patient was homoplasmic for the mutation m.10663T>C p.ND4L: (Val65AIa). At initial diagnosis the patient presents with thinning of the RNFL in the left eye in the superior and nasal quadrant. At follow up there is significant reduction of RNFL thickness bilaterally, more seen in the superior and nasal quadrant and is more evident in the left eye. *OD, Oculus dexter; OS, Oculus sinister; CF, Counting fingers; RNFL, Retinal nerve fiber layer.*

**Figure 4:**
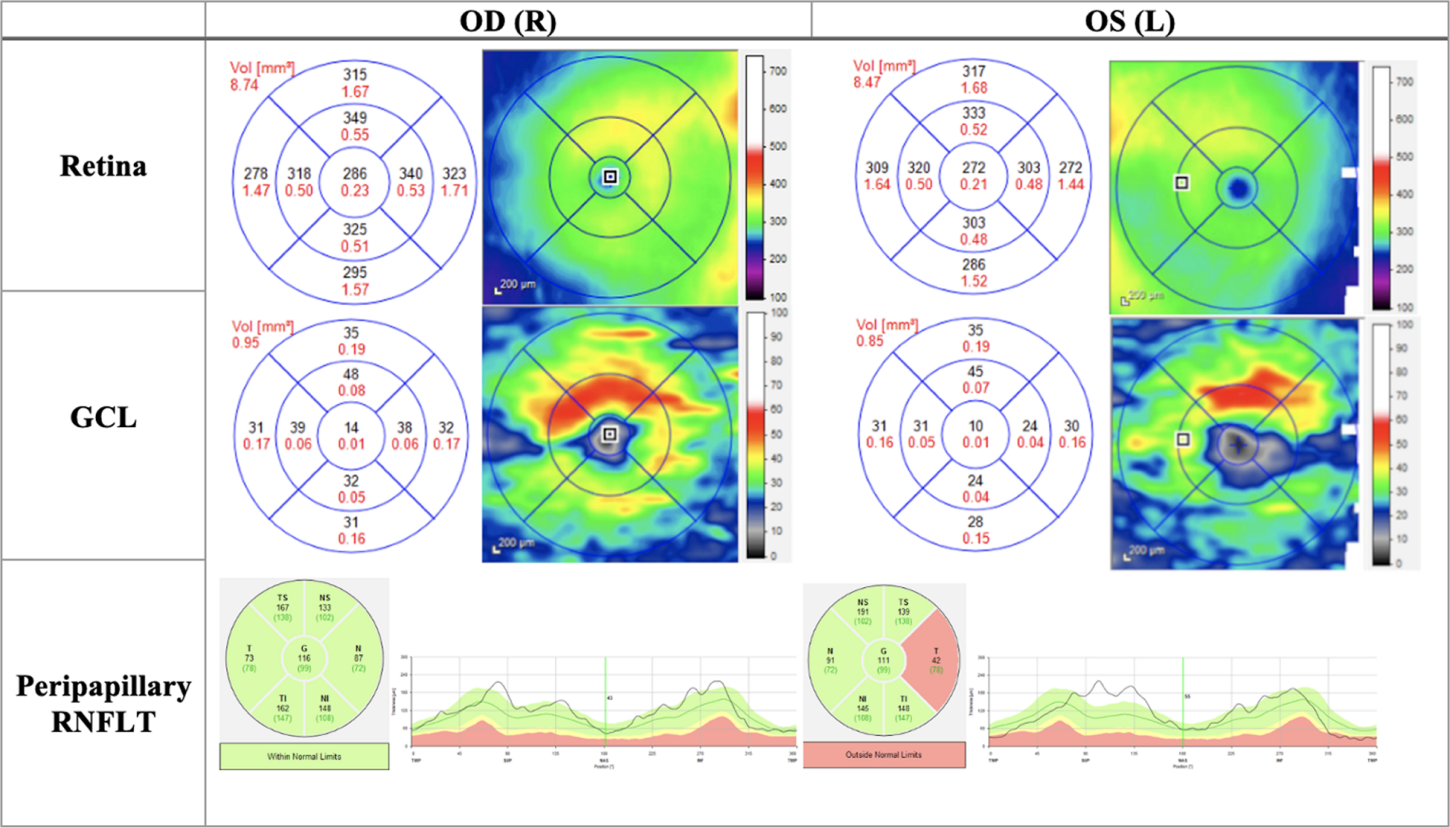
OCT (Heidelberg, Spectralis) findings of patient B, a 20-year-old male patient presenting to the clinic with bilateral vision loss and bilateral central scotomas of 3-month duration. The thickness map shows thinning of GCL despite normal RNFLT in OD, and thinning of the temporal RNFT with corresponding GCL thinning in OS. *OD, Oculus dexter; OS, Oculus sinister; GCL, Ganglion cell layer; RNFLT, Retinal nerve fiber layer thickness.*

**Figure 5:**
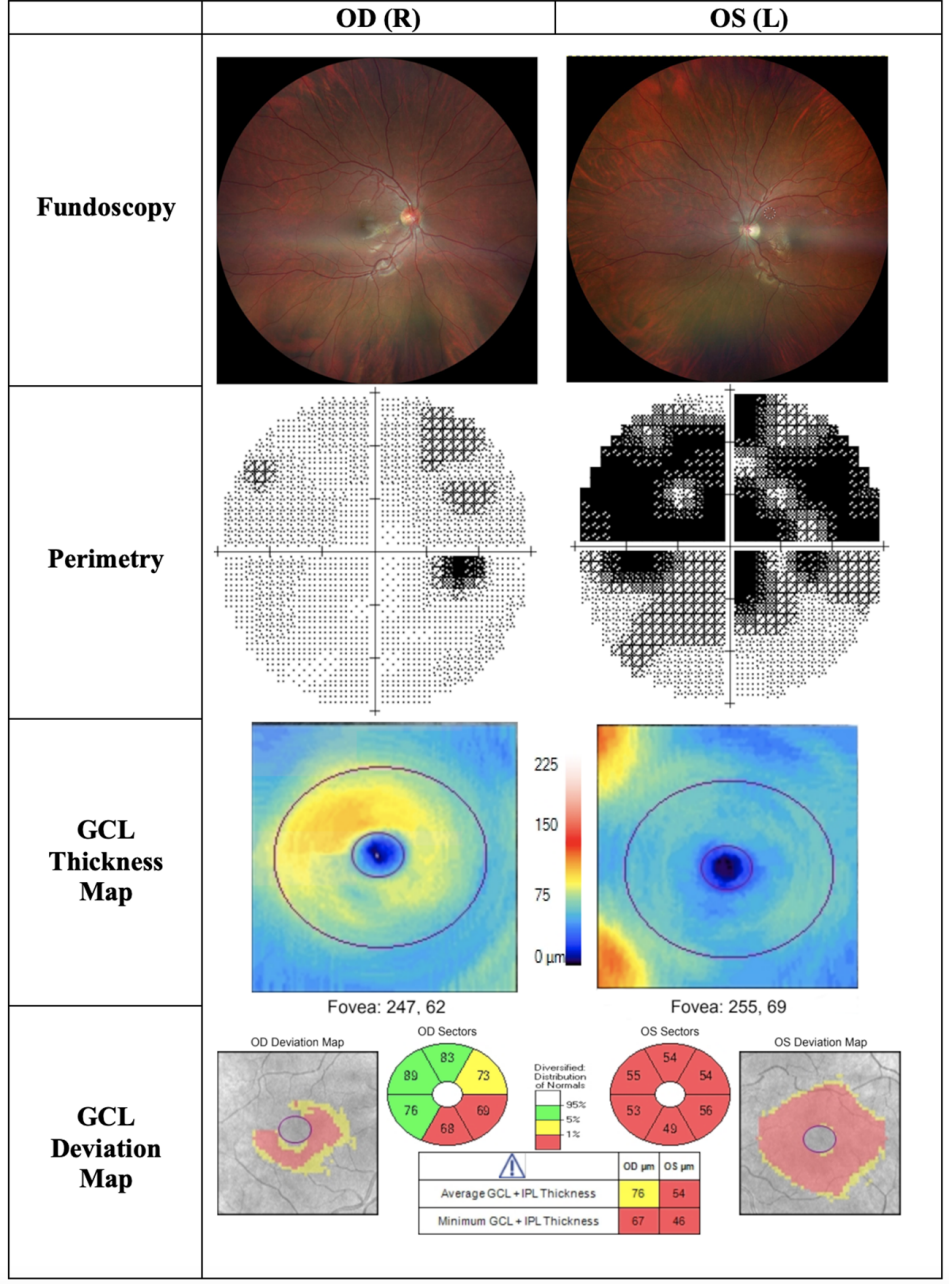
Findings of patient C, a 22-year-old male patient presenting to the clinic with progressive central visual loss OS for the duration of 5 months. His visual acuity was 2/200 OS and 20/400 OD. Fundoscopy showed a pale disc OS and peripapillary telangiectasias OD. The thickness map shows marked GCL thinning OS and early thinning of GCL OD, corresponding with deterioration of visual acuity. *OD, Oculus dexter; OS, Oculus sinister; OU, Oculus uterque; GCL, Ganglion cell layer.*

In the acute phase, patients present with symptoms of a central or ceco-central scotoma, and visual acuity can deteriorate significantly with dyschromatopsia and reduced contrast sensitivity (48). Specifically, protan and tritan colour sensitivity are affected early on in LHON (49). Fundus examination may show vascular tortuosity, pseudo-edema, optic disc hyperemia, or peripapillary telangiectasis (50). However, it is not uncommon for the fundus examination to be normal (20-40% of patients), which can delay the diagnosis (Yu-Wai-Man et al., (51)) (**Figure 2**). Spectral-domain OCT at this stage will show thickening of the inferior and superior RNFL, which is synchronous with thinning of the inferior-temporal RNFL. At the onset of visual deterioration, the GCL and the inner plexiform layer undergo significant reduction in thickness, which later correlates with further deterioration in vision (46, 52). As the acute phase progresses, RNFL swelling normalises (45) (**Figures 3**, **4**, **5**). Furthermore, early abnormalities after the onset of symptoms can be detected using visual evoked potentials (VEPs), including an absent response, decreased amplitudes, and delayed latency (40). ERG can show reduced cone-responses and N50-90 amplitudes (53). Furthermore, MRI findings have been reported in the early stages of LHON and include optic nerve enhancement in post-contrast MRI images (54).

The chronic phase is characterised by optic atrophy, which can develop as soon as six weeks from initial clinical presentation (55, 56). Patients at this stage reach a plateau of visual deterioration, and their chance for visual recovery is diminished. OCT will show GCL thinning, which is well established in the chronic phase, and RNFL thinning is also evident (**Figure 5**) (45). The progression in OCT findings from the pre-symptomatic phase to early and late acute, and eventually the chronic phase, is essential in following up patient progression in LHON.

Patients with LHON may manifest extraocular features (LHON plus syndromes), including cardiac arrhythmias, such as Wolff-Parkinson-White syndrome. Therefore an EKG is recommended in the comprehensive clinical evaluation of LHON patients (57) (58). Neurological features may include peripheral neuropathy, postural tremors, clonus, dystonia, non-specific myopathy, and movement disorders. Therefore, a complete neurological exam and a brain MRI may be warranted in some cases of LHON (59, 60). In addition, neuro-psychiatric disturbances, spastic dystonia, ataxia, and juvenile-onset encephalopathy have been reported in some cases of LHON (61, 62). Furthermore, the LHON-Multiple sclerosis phenotype (Harding syndrome) is a LHON-plus syndrome that can be difficult to distinguish from multiple sclerosis. This syndrome can present with an optic neuritis-like picture (ocular pain with bilateral vision loss), disseminated central nervous system demyelination, periventricular white matter lesions, and positive oligoclonal bands in the cerebrospinal fluid (63).

Leigh syndrome is a rare neurodegenerative mitochondrial disorder most commonly affecting children aged three to twelve months, but it can infrequently be observed in adulthood (64). It is characterized by psychomotor regression, peripheral neuropathy, cerebellar ataxia, spasticity, and hypotonia (65). Ocular manifestations include nystagmus, ophthalmoparesis, and optic atrophy (66). Leigh syndrome has been reported in association with LHON-phenotype through MT-ND6 mutations, which include G14459A and T14484C point mutations (67).

The diagnosis of LHON is based on clinical presentation with the exclusion of alternative etiologies (optic neuritis, compressive, or toxic optic neuropathy), the results of ancillary tests (visual field, OCT, VEP, ERG), and confirmation by molecular genetic testing. Genetic testing can be initially targeted at the three common pathogenic types of LHON, followed by a multi-gene panel for mitochondrial diseases, including NADH dehydrogenase. Finally, if both yield negative results, complete mtDNA sequencing is performed (51, 68). In a patient with a positive family history of LHON with typical symptoms, genetic testing may not be required for diagnosis, but confirmation of the underlying mutation may be prognostically valuable (51). In addition, *de novo* mutations can possibly arise in coherence with an established pedigree.

## Management and therapeutic approach

As with the majority of mitochondrial disorders, the therapeutic options for LHON remain only supportive. It is essential to counsel patients about the deleterious effects of smoking, the consumption of large amounts of alcohol, and certain medications and toxins that can adversely affect mitochondrial function. Furthermore, low vision rehabilitation and aids can be an option in patients with intact peripheral vision (69). Younger patients with the onset of disease at the age of less than 20 years have been reported to have a better visual prognosis (70). In addition, a subacute course of vision loss, as well as a larger optic disc, are both favourable prognostic indicators for visual recovery (71, 72).

Ubiquinone analogues such as co-enzyme Q10 function as carriers of electrons from complex I to complex II of the respiratory chain. However, evidence for the clinical benefits of co-enzyme Q10 in LHON patients is lacking. In addition, the lipophilic nature of the compound makes it poorly absorbable across the intestinal tract (73, 74). On the other hand, idebenone is a short-chained water-soluble ubiquinone that is easily absorbed through the oral route. It provides protection by bypassing complex I, maintaining ATP production, and protecting against mitochondrial oxidative damage (75). Idebenone has been found to be beneficial in promoting vision recovery in LHON patients, particularly in the early stages of the disease and in younger patients (49, 76–78). Currently, idebenone is approved by the European Medicines Agency for the treatment of LHON in adolescent and adult patients at a dose of 900mg/day divided into three doses. Treatment should be continued for at least a year or until a plateau of vision improvement is reached (79) (**Table 1**). The “Post-Authorisation Safety Study with Raxone^®^ in LHON Patients” was completed in 2021 (NCT02771379). Another ubiquinone analogue with *in vitro* activity superior to idebenone, EPI-743, is in the experimental phase and has shown potential benefits in LHON visual recovery (80).

**Table 1:** Idebenone in the treatment of LHON.

Study design	End-points	Summary Points
**RHODOS Trial NCT00747487** **(** 78**)** *24-week double-blinded RCT.* *Idebenone 900mg/day, n=55.* *Placebo, n=30.*	-Primary: Best recovery of visual acuity between baseline and Week 24. -Secondary: Change from baseline to Week 24 in best visual acuity. Change in visual acuity of the best eye at baseline. Change in visual acuity for both eyes in each patient. Acuity assessed with: ETDRS charts.	Main Findings: -Statistical significance not reached in the primary end point. -Beneficial effect of idebenone over placebo present when assessing all secondary end-points. Supplementary Findings: -Largest treatment effects in m.11778G>A and m.3460G>A mutations.
**Idebenone Treatment In Leber’s Hereditary Optic Neuropathy** **(** 76**)** *Retrospective evaluation of idebenone therapy.* *Idebenone cohort, n=44* *Untreated control cohort, n=59*	-Primary: Recovery of visual acuity defined as a gain of at least two lines on Snellen acuity or a change from ‘off chart’ to ‘on chart’.	Main Findings: -Increased frequency of recovery was significant with the use of idebenone in m.11778G>A patients. -Early start of therapy was the most predictive factor for visual recovery. Supplementary Findings: -Trend for earlier onset of visual recovery in treated patients compared with untreated.
**RHODOS–OFU** **NCT01421381** **(** 77**)** *Single visit observational follow-up study 30 months following the end of the RHODOS trial.* *Idebenone 900mg/day, n=39.* *Placebo, n=19.*	-Primary: Change in best visual acuity assessed at this study visit compared with baseline and Week 24 of RHODOS.	Main Findings: -Beneficial effects from 24 weeks of treatment with idebenone during RHODOS persisted despite discontinuation of therapy for a median time of 30 months.

Various vitamins and supplements, such as vitamin B_12_, vitamin C, vitamin E, thiamine, riboflavin, L-carnitine, L-arginine, and creatine, have been used in LHON patients.

The presence of vitamin B_12_ deficiency was statistically significant for LHON mutation carriers in the general population, and excess alcohol consumption was a significant predictor of such deficiency (81). However, despite the safety profile of these various vitamins and supplements, there were no proven clinical benefits for promoting visual recovery in LHON patients (82).

Brimonidine, a topical α2-agonist used to manage glaucoma patients, has shown protective anti-apoptotic value in RGCs in animal models (83). Unfortunately, when used in LHON patients, brimonidine did not appear to be efficacious (84). Nonetheless, its use in LHON patients with concurrent glaucoma may be justified.

Gene therapy is the latest therapeutic strategy for LHON that has shown some promising results. Currently, gene therapy in LHON aims to deliver the un-mutated MT-ND4 gene into RGC nuclei with the goal of producing functioning proteins/complex I subunits that can be embedded into the mitochondrial respiratory chain. Recombinant adeno-associated viral vector rAAV2, which encodes human wild-type MT-ND4, has been used in multiple trials and proved to be a valuable contribution to LHON treatment.

The use of the viral vector rAAV2-ND4 was first introduced in a trial by Wan et al. in 2010. Nine patients were enrolled in a phase 1 trial (NCT01267422). Eight of those enrolled received an intravitreal injection of the vector in one eye, while one patient received the vector in both eyes. Six of the nine patients exhibited an improvement in BCVA of at least 0.3 logMAR after a period of nine months (85). In 2017, a group of 149 patients was recruited by the same Wuhan research group for an interventional trial where they received a single unilateral intravitreal injection of rAAV2-ND4 (NCT03153293). Within three days, 54 patients exhibited significant improvement in VA of more than 0.3 logMAR in at least one eye (86). Furthermore, a single unilateral injection was found to result in bilateral visual acuity improvement (0.21 logMAR treated eye; 0.24 logMAR untreated eye)12 months post-therapy (87).

GS010, which is a recombinant adeno-associated viral vector serotype 2 (rAAV2) that encodes human wild-type MT-ND4, has shown improved visual acuity when injected into the vitreous cavity of a single eye during clinical trials carried out by GenSight Biologics (88). In 2017, in two phase III clinical trials *rescue* (NCT02652767) (LHON patients with vision loss <6 months) and *reverse* (NCT02652780) (LHON patients with vision loss >6 months to 1 year), GS010 was randomly injected into one eye, while the other eye received a sham injection. In these trials, patients experienced significant improvement in visual acuity in the treated eyes as well as the sham eyes, raising the possibility of possible vector transfer from the GS010 eye to the sham eye (39). 71% of *rescue* and 76% of *reverse* patients had at least 0.3 gain of logMAR VA in at least one eye. In addition, a clinically relevant recovery at week 96 post-treatment was seen in 71% of *rescue* and 81% of *reverse* patients (89). In the ongoing phase III *reflect* trial (NCT03293524), GS010 was injected bilaterally in subjects with LHON exhibiting the m.11778G>A mutation when vision loss was present for less than one year and showed greater efficacy in visual recovery of +5 ETDRS when compared to GS010 injected in a single eye (90) (**Table 2**).

**Table 2:** GS010 in the treatment of LHON.

Study design	End-points	Summary Points
**RESCUE Trial** **NCT02652767 (** 89**)** ***Double-blinded RCT.*** ***LHON patients (m.11778G>A) with vision loss of 6 months or less.*** ***Each participant had one eye randomly selected to receive GS010 and the other eye received a sham injection.*** ***Intravitreal injection of a single dose of GS010 in one eye.*** ***n=39.*** ***Sham injection in the other eye.*** ***n=39.***	-Primary: Difference in change from baseline in ETDRS Visual Acuity at Week 48 between GS010 and sham. -Secondary: Difference in change from baseline in ETDRS visual acuity at Week 72 & 96 between GS010 and sham. Number of eye responders (15 letter ETDRS improvement vs baseline) to treatment. Number of Subject Responders (15 letter ETDRS improvement compared to sham in same patient) to treatment.	Main Findings: - 71% of subjects had an improvement of at least -0.3 logMAR (15 ETDRS letters equivalent) from the nadir in at least one eye. -Improvement from nadir is significant ( P < 0.0001) and occurred at similar magnitude in both eyes. -Bilateral improvement in vision occurred after a nadir of deterioration at week 24. -Primary end point of -0.3 logMar (15-letter) was not met due to bilateral improvement.
**REVERSE Trial** **NCT02652780 (** 91**)** ***Double-blinded RCT.*** ***LHON patients (m.11778G>A) with vision loss of more than 6 months and up to 1 year.*** ***Each participant had one eye randomly selected to receive GS010 and the other eye received a sham injection.*** ***Intravitreal injection of a single dose of GS010 in one eye.*** ***n=37.*** ***Sham injection in the other eye.*** ***n=37.***	-Primary: Difference in change from baseline in ETDRS Visual Acuity at Week 48 between GS010 and sham. -Secondary: Difference in change from baseline in ETDRS visual acuity at Week 72 & 96 between GS010 and sham. Number of eye responders (15 letter ETDRS improvement vs baseline) to treatment. Number of Subject Responders (15 letter ETDRS improvement compared to sham in same patient) to treatment.	Main Findings: - 76% of subjects had an improvement of at least -0.3 logMAR (15 ETDRS letters equivalent) from the nadir in at least one eye. -Bilateral improvement in vision occurred after a nadir of deterioration at week 12. -Primary end point of -0.3 logMar (15-letter) was not met due to bilateral improvement.
**REFLECT Trial** **NCT03293524 (** 90**)** ***ONGOING TRIAL*** ***Double-blinded RCT.*** ***LHON patients (m.11778G>A) with vision loss of up to 1 year.*** ***Each participant received GS010 in their first-affected eye, and either gene therapy or placebo in their second-affected eye.*** ***Intravitreal GS010 in both eyes.*** ***n=48.*** ***GS010 in one eye and placebo intravitreal injection in the other eye.*** ***n=50.***	-Primary: BCVA in 2^nd^ affected eye reported with LogMar from baseline at 1.5 years. -Secondary: BCVA in 2nd affected eye reported with LogMar from baseline at 2 years.	Main Findings: -Average final visual acuity was reported in subjects treated bilaterally, compared to subjects treated unilaterally was +5 ETDRS letters. Supplementary Findings: -BCVA improvement between second-affected eyes was equivalent to +3 ETDRS letters in favor of GS010 at 1.5 years. -2^nd^ affected eyes treated with GS010 showed +19 ETDRS letters improvement over nadir (p<0.0001) at 1.5 years. -2^nd^ affected eyes receiving placebo showed +16 ETDRS letters improvement over nadir (p<0.0001) at 1.5 years.

**Figure 6:**
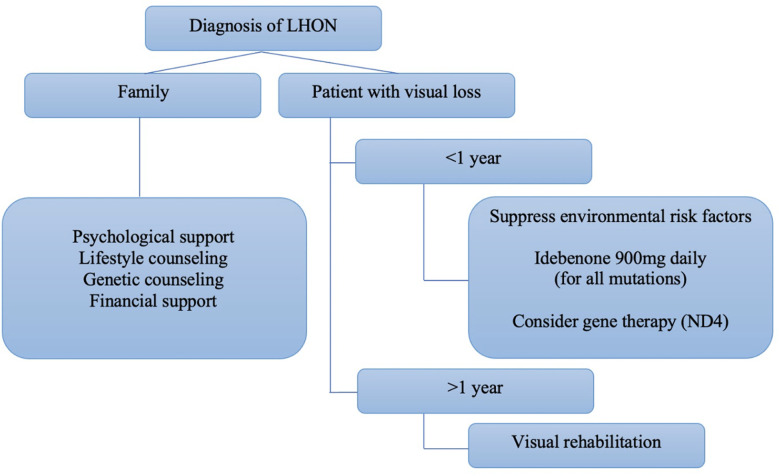
Algorithm proposed by Hage and Vignal-Clermont considering the statements of the Milan panel and gene therapy as part of a treatment guide for LHON (Hage & Vignal-Clermont, (39)).

An expert panel consensus on the therapeutic management of LHON recommended the use of idebenone at a dose of 900mg/day for at least one year as the first-line treatment for patients with less than one year since the onset of the disease. However, there was no evidence to recommend treatment for chronic cases (more than one year since the onset of symptoms in the second eye) (**Figure 6**). Gene therapy was not included in the panel’s recommendation (39).

## Screening and genetic counselling

If a primary LHON mutation is detected in a proband, screening of other family members can be offered to exclude the possibility of a *de novo* mutation (92). Given that LHON is maternally inherited, all males should be reassured that none of their offspring will inherit the mutation. On the other hand, homoplasmic females will transmit the mutation to all their offspring. In heteroplasmic mothers, varying levels of mutant mtDNA is transferred to offspring, with approximately 60% mutant mtDNA required as a threshold for disease manifestation (23). This, however, should be cautiously discussed with patients as the presence of a clear threshold is variable.

Children of homoplasmic mothers need to be aware that the penetrance of LHON is variable, and not all carriers develop the disease. Risk stratification for penetrance in carriers can be assessed by considering their age, gender, and other prognostic factors (10). Age and gender appear to be the most critical factors in evaluating the risk of penetrance: however, patients must be aware that these predictions are still estimates. Carriers should also avoid smoking and heavy alcohol consumption, as well as possible triggers for the disease.

## Conclusion

LHON is a mitochondrial optic neuropathy that affects young males but is also not uncommon in females. Recently, advancements have been made in understanding the pathophysiology of LHON and developing new therapeutic strategies, such as gene therapy through the use of viral vectors in clinical trials. However, further studies are required to incorporate gene therapy as a universally approved treatment for LHON.

## Author contributions

AE contributed to the literature search and writing of the manuscript and abstract. AA contributed to writing of the manuscript and illustrations. RB contributed by supervising the review, providing appropriate modifications to the manuscript, and providing expert opinion. All authors contributed to the article and approved the submitted version.

## References

[1] Sundaramurthy S SelvaKumar A Ching J Dharani V Sarangapani S Yu-Wai-Man P. Leber hereditary optic neuropathy-new insights and old challenges. Graefes Arch Clin Exp Ophthalmol (2021) 259(9):2461–72. doi: 10.1007/s00417-020-04993-1 33185731

[2] Carper MG Henderson AD. Updated review of leber hereditary optic neuropathy. Curr Treat Options Neurol (2022) 24(9):441–52. doi: 10.1007/s11940-022-00729-0

[3] Leber T. Ueber hereditäre und congenital-angelegte Sehnervenleiden. Albrecht von Graefes Archiv für Ophthalmologie (1871) 17(2):249–91. doi: 10.1007/BF01694557

[4] Leber T. Ueber hereditäre und congenital-angelegte sehnervenleiden. Albrecht von Graefes Archiv für Ophthalmologie (1871) 17(2):249–91. doi: 10.1007/BF01694557

[5] Wallace DC Singh G Lott MT Hodge JA Schurr TG Lezza AM. Mitochondrial DNA mutation associated with leber's hereditary optic neuropathy. Science (1988) 242(4884):1427–30. doi: 10.1126/science.3201231 3201231

[6] Yu-Wai-Man P Griffiths PG Brown DT Howell N Turnbull DM Chinnery PF. The epidemiology of leber hereditary optic neuropathy in the north East of England. Am J Hum Genet (2003) 72(2):333–9. doi: 10.1086/346066 PMC379226 12518276

[7] Schapira AHV. Mitochondrial disease. Lancet (2006) 368(9529):70–82. doi: 10.1016/S0140-6736(06)68970-8 16815381

[8] Puomila A Hämäläinen P Kivioja S Savontaus ML Koivumäki S Huoponen K. Epidemiology and penetrance of leber hereditary optic neuropathy in Finland. Eur J Hum Genet (2007) 15(10):1079–89. doi: 10.1038/sj.ejhg.5201828 17406640

[9] Rosenberg T Nørby S Schwartz M Saillard J Magalhães PJ Leroy D. Prevalence and genetics of leber hereditary optic neuropathy in the Danish population. Invest Ophthalmol Visual Sci (2016) 57(3):1370–5. doi: 10.1167/iovs.15-18306 27007794

[10] Lopez Sanchez MIG Kearns LS Staffieri SE Clarke L McGuinness MB Meteoukki W. Establishing risk of vision loss in leber hereditary optic neuropathy. Am J Hum Genet (2021) 108(11):2159–70. doi: 10.1016/j.ajhg.2021.09.015 PMC8595929 34670133

[11] Takano F Ueda K Godefrooij DA Yamagami A Ishikawa H Chuman H. Incidence of leber hereditary optic neuropathy in 2019 in Japan: a second nationwide questionnaire survey. Orphanet J Rare Dis (2022) 17(1):319. doi: 10.1186/s13023-022-02478-4 35987635 PMC9392235

[12] Poincenot L Pearson AL Karanjia R. Demographics of a Large international population of patients affected by leber's hereditary optic neuropathy. Ophthalmology (2020) 127(5):679–88. doi: 10.1016/j.ophtha.2019.11.014 31932089

[13] Chinnery PF Hudson G. Mitochondrial genetics. Br Med Bull (2013) 106(1):135–59. doi: 10.1093/bmb/ldt017 PMC3675899 23704099

[14] Procaccio V Mousson B Beugnot R Duborjal H Feillet F Putet G. Nuclear DNA origin of mitochondrial complex I deficiency in fatal infantile lactic acidosis evidenced by transnuclear complementation of cultured fibroblasts. J Clin Invest (1999) 104(1):83–92. doi: 10.1172/JCI6184 10393702 PMC408404

[15] Prasun P. Chapter 10 - mitochondrial DNA point mutation disorders. In: Prasun P, editor. Mitochondrial medicine. New York, NY, United States: Academic Press (2019). p. 37–47.

[16] Tonagel F Wilhelm H Richter P Kelbsch C. Leber’s hereditary optic neuropathy: course of disease in consideration of idebenone treatment and type of mutation. Graefe's Arch Clin Exp Ophthalmol (2021) 259(4):1009–13. doi: 10.1007/s00417-020-05045-4 PMC8016777 33337510

[17] Macmillan C Kirkham T Fu K Allison V Andermann E Chitayat D. Pedigree analysis of French Canadian families with T14484C leber's hereditary optic neuropathy. Neurology (1998) 50(2):417–22. doi: 10.1212/WNL.50.2.417 9484365

[18] Mackey DA Oostra RJ Rosenberg T Nikoskelainen E Bronte-Stewart J Poulton J. Primary pathogenic mtDNA mutations in multigeneration pedigrees with leber hereditary optic neuropathy. Am J Hum Genet (1996) 59(2):481–5. PMC1914749 8755941

[19] Harding AE Sweeney MG Govan GG Riordan-Eva P. Pedigree analysis in leber hereditary optic neuropathy families with a pathogenic mtDNA mutation. Am J Hum Genet (1995) 57(1):77–86. 7611298 PMC1801226

[20] Johns DR Heher KL Miller NR Smith KH. Leber's hereditary optic neuropathy. clinical manifestations of the 14484 mutation. Arch Ophthalmol (1993) 111(4):495–8. doi: 10.1001/archopht.1993.01090040087038 8470982

[21] Johns DR Smith KH Miller NR. Leber's hereditary optic neuropathy. clinical manifestations of the 3460 mutation. Arch Ophthalmol (1992) 110(11):1577–81. doi: 10.1001/archopht.1992.01080230077025 1444915

[22] Smith KH Johns DR Heher KL Miller NR. Heteroplasmy in leber's hereditary optic neuropathy. Arch Ophthalmol (1993) 111(11):1486–90. doi: 10.1001/archopht.1993.01090110052022 8240102

[23] Chinnery PF Andrews RM Turnbull DM Howell NN. Leber hereditary optic neuropathy: Does heteroplasmy influence the inheritance and expression of the G11778A mitochondrial DNA mutation? Am J Med Genet (2001) 98(3):235–43. doi: 10.1002/1096-8628(20010122)98:3<235::AID-AJMG1086>3.0.CO;2-O 11169561

[24] Bianco A Bisceglia L Trerotoli P Russo L D'Agruma L Guerriero S. Leber's hereditary optic neuropathy (LHON) in an apulian cohort of subjects. Acta Myol (2017) 36(3):163–77. PMC5953227 29774306

[25] Mitchell SL Goodloe R Brown-Gentry K Pendergrass SA Murdock DG Crawford DC. Characterization of mitochondrial haplogroups in a large population-based sample from the united states. Hum Genet (2014) 133(7):861–8. doi: 10.1007/s00439-014-1421-9 PMC4113317 24488180

[26] Man PYW Turnbull DM Chinnery PF. Leber hereditary optic neuropathy. J Med Genet (2002) 39(3):162. doi: 10.1136/jmg.39.3.162 11897814 PMC1735056

[27] Dai Y Wang C Nie Z Han J Chen T Zhao X. Mutation analysis of leber's hereditary optic neuropathy using a multi-gene panel. BioMed Rep (2018) 8(1):51–8. doi: 10.3892/br.2017.1014 PMC5768074 29387390

[28] Yum HR Chae H Shin SY Kim Y Kim M Park SH. Pathogenic mitochondrial DNA mutations and associated clinical features in Korean patients with leber's hereditary optic neuropathy. Invest Ophthalmol Visual Sci (2014) 55(12):8095–101. doi: 10.1167/iovs.14-15311 25342614

[29] Giordano C Montopoli M Perli E Orlandi M Fantin M Ross-Cisneros FN. Oestrogens ameliorate mitochondrial dysfunction in leber's hereditary optic neuropathy. Brain (2011) 134(Pt 1):220–34. doi: 10.1093/brain/awq276 PMC3025718 20943885

[30] Yu J Liang X Ji Y Ai C Liu J Zhu L. PRICKLE3 linked to ATPase biogenesis manifested leber's hereditary optic neuropathy. J Clin Invest (2020) 130(9):4935–46. doi: 10.1172/JCI134965 PMC7456240 32516135

[31] Yu-Wai-Man P Griffiths PG Chinnery PF. Mitochondrial optic neuropathies - disease mechanisms and therapeutic strategies. Prog Retin Eye Res (2011) 30(2):81–114. doi: 10.1016/j.preteyeres.2010.11.002 21112411 PMC3081075

[32] Kirkman MA Yu-Wai-Man P Korsten A Leonhardt M Dimitriadis K De Coo IF. Gene-environment interactions in leber hereditary optic neuropathy. Brain (2009) 132(Pt 9):2317–26. doi: 10.1093/brain/awp158 PMC2732267 19525327

[33] Giordano L Deceglie S d'Adamo P Valentino ML La Morgia C Fracasso F. Cigarette toxicity triggers leber's hereditary optic neuropathy by affecting mtDNA copy number, oxidative phosphorylation and ROS detoxification pathways. Cell Death Dis (2015) 6(12):e2021. doi: 10.1038/cddis.2015.364 26673666 PMC4720897

[34] Nolfi-Donegan D Braganza A Shiva S. Mitochondrial electron transport chain: Oxidative phosphorylation, oxidant production, and methods of measurement. Redox Biol (2020) 37:101674. doi: 10.1016/j.redox.2020.101674 32811789 PMC7767752

[35] Sadun A Carelli V La Morgia C Karanjia R. Leber's hereditary optic neuropathy (LHON) mtDNA mutations cause cell death by overproduction of reactive oxygen species. Acta Ophthalmologica (2015) 93(S255). doi: 10.1111/j.1755-3768.2015.0131

[36] Qi X Lewin AS Hauswirth WW Guy J. Optic neuropathy induced by reductions in mitochondrial superoxide dismutase. Invest Ophthalmol Visual Sci (2003) 44(3):1088–96. doi: 10.1167/iovs.02-0864 12601034

[37] Kinnally KW Peixoto PM Ryu SY Dejean LM. Is mPTP the gatekeeper for necrosis, apoptosis, or both? Biochim Biophys Acta (2011) 1813(4):616–22. doi: 10.1016/j.bbamcr.2010.09.013 PMC3050112 20888866

[38] Bargiela D Chinnery PF. Mitochondria in neuroinflammation – multiple sclerosis (MS), leber hereditary optic neuropathy (LHON) and LHON-MS. Neurosci Lett (2019) 710:132932. doi: 10.1016/j.neulet.2017.06.051 28668384

[39] Hage R Vignal-Clermont C. Leber hereditary optic neuropathy: Review of treatment and management. Front Neurol (2021) 12:651639. doi: 10.3389/fneur.2021.651639 34122299 PMC8187781

[40] Majander A Robson AG João C Holder GE Chinnery PF Moore AT. The pattern of retinal ganglion cell dysfunction in leber hereditary optic neuropathy. Mitochondrion (2017) 36:138–49. doi: 10.1016/j.mito.2017.07.006 PMC5644721 28729193

[41] Shemesh A Sood G Margolin E. Leber hereditary optic neuropathy (LHON). Treasure Island (FL: StatPearls (2022). 29494105

[42] Sadun AA Salomao SR Berezovsky A Sadun F Denegri AM Quiros PA. Subclinical carriers and conversions in leber hereditary optic neuropathy: a prospective psychophysical study. Trans Am Ophthalmol Soc (2006) 104:51–61. 17471325 PMC1809912

[43] Moura AL Nagy BV La Morgia C Barboni P Oliveira AG Salomão SR. The pupil light reflex in leber's hereditary optic neuropathy: evidence for preservation of melanopsin-expressing retinal ganglion cells. Invest Ophthalmol Vis Sci (2013) 54(7):4471–7. doi: 10.1167/iovs.12-11137 PMC4322722 23737476

[44] Do MT Yau KW. Intrinsically photosensitive retinal ganglion cells. Physiol Rev (2010) 90(4):1547–81. doi: 10.1152/physrev.00013.2010 PMC4374737 20959623

[45] Hedges TR Gobuty M Manfready RA Erlich-Malona N Monaco C Mendoza-Santiesteban CE. The optical coherence tomographic profile of leber hereditary optic neuropathy. Neuroophthalmology (2016) 40(3):107–12. doi: 10.3109/01658107.2016.1173709 PMC5123139 27928393

[46] Carbonelli M La Morgia C Romagnoli M Amore G D'Agati P Valentino ML. Capturing the pattern of transition from carrier to affected in leber hereditary optic neuropathy. Am J Ophthalmol (2022) 241:71–9. doi: 10.1016/j.ajo.2022.04.016 35513027

[47] Barboni P Savini G Feuer WJ Budenz DL Carbonelli M Chicani F. Retinal nerve fiber layer thickness variability in leber hereditary optic neuropathy carriers. Eur J Ophthalmol (2012) 22(6):985–91. doi: 10.5301/ejo.5000154 22562299

[48] Kirkman MA Korsten A Leonhardt M Dimitriadis K De Coo IF Klopstock T. Quality of life in patients with leber hereditary optic neuropathy. Invest Ophthalmol Vis Sci (2009) 50(7):3112–5. doi: 10.1167/iovs.08-3166 19255150

[49] Rudolph G Dimitriadis K Büchner B Heck S Al-Tamami J Seidensticker F. Effects of idebenone on color vision in patients with leber hereditary optic neuropathy. J Neuroophthalmol (2013) 33(1):30–6. doi: 10.1097/WNO.0b013e318272c643 PMC3658961 23263355

[50] Khanh Vu TH Zhu R Yang L Chen DF. Optic Nerve Structure and Pathologies. In: McManus LM Mitchell RN, editors. Pathobiology of Human Disease (San Diego: Academic Press) (2014) 2115–25.

[51] Yu-Wai-Man P Griffiths PG Hudson G Chinnery PF. Inherited mitochondrial optic neuropathies. J Med Genet (2009) 46(3):145–58. doi: 10.1136/jmg.2007.054270 PMC2643051 19001017

[52] Moster SJ Moster ML Scannell Bryan M Sergott RC. Retinal ganglion cell and inner plexiform layer loss correlate with visual acuity loss in LHON: A longitudinal, segmentation OCT analysis. Invest Ophthalmol Vis Sci (2016) 57(8):3872–83. doi: 10.1167/iovs.15-17328 27459664

[53] Ziccardi L Sadun F De Negri AM Barboni P Savini G Borrelli E. Retinal function and neural conduction along the visual pathways in affected and unaffected carriers with Leber’s hereditary optic neuropathy. Invest Ophthalmol Vis Sci (2013) 54(10):6893–901. doi: 10.1167/iovs.13-12894 24071953

[54] Vaphiades MS Phillips PH Turbin RE. Optic nerve and chiasmal enhancement in leber hereditary optic neuropathy. J Neuro-Ophthalmology (2003) 23(1). doi: 10.1097/00041327-200303000-00057 12616096

[55] Borrelli E Triolo G Cascavilla ML La Morgia C Rizzo G Savini G. Changes in choroidal thickness follow the RNFL changes in leber’s hereditary optic neuropathy. Sci Rep (2016) 6(1):37332. doi: 10.1038/srep37332 27853297 PMC5112509

[56] Lin Y-H Wang N-K Yeung L Lai C-C Chuang L-H. Juvenile open-angle glaucoma associated with leber’s hereditary optic neuropathy: a case report and literature review. BMC Ophthalmol (2018) 18(1):323. doi: 10.1186/s12886-018-0980-2 30558558 PMC6296145

[57] Sorajja P Sweeney MG Chalmers R Sachdev B Syrris P Hanna M. Cardiac abnormalities in patients with leber's hereditary optic neuropathy. Heart (2003) 89(7):791–2. doi: 10.1136/heart.89.7.791 PMC1767718 12807863

[58] Finsterer J Stöllberger C Kopsa W Jaksch M. Wolff-Parkinson-White syndrome and isolated left ventricular abnormal trabeculation as a manifestation of leber's hereditary optic neuropathy. Can J Cardiol (2001) 17(4):464–6. 11329546

[59] Nikoskelainen EK Marttila RJ Huoponen K Juvonen V Lamminen T Sonninen P. Leber's "plus": neurological abnormalities in patients with leber's hereditary optic neuropathy. J Neurol Neurosurg Psychiatry (1995) 59(2):160–4. doi: 10.1136/jnnp.59.2.160 PMC485991 7629530

[60] Martikainen MH Ng YS Gorman GS Alston CL Blakely EL Schaefer AM. Clinical, genetic, and radiological features of extrapyramidal movement disorders in mitochondrial disease. JAMA Neurol (2016) 73(6):668–74. doi: 10.1001/jamaneurol.2016.0355 27111573

[61] Berardo A Emmanuele V Vargas W Tanji K Naini A Hirano M. Leber hereditary optic neuropathy plus dystonia, and transverse myelitis due to double mutations in MT-ND4 and MT-ND6. J Neurol (2020) 267(3):823–9. doi: 10.1007/s00415-019-09619-z PMC7362294 31776719

[62] Funalot B Reynier P Vighetto A Ranoux D Bonnefont J-P Godinot C. Leigh-Like encephalopathy complicating leber's hereditary optic neuropathy. Ann Neurol (2002) 52(3):374–7. doi: 10.1002/ana.10299 12205655

[63] Matthews L Enzinger C Fazekas F Rovira A Ciccarelli O Dotti M. MRI In leber's hereditary optic neuropathy:The relationship to multiple sclerosis. J neurology neurosurgery Psychiatry (2014) 86. doi: 10.1136/jnnp-2014-308186 PMC4413690 25053773

[64] Jabeen SA Sandeep G Mridula KR Meena AK Borgohain R Sundaram C. Adult-onset leigh's disease: A rare entity. Ann Indian Acad Neurol (2016) 19(1):140–2. doi: 10.4103/0972-2327.175437 PMC4782535 27011650

[65] Thorburn DR Rahman J Rahman S. Mitochondrial DNA-associated Leigh syndrome and NARP. In: Adam MP Everman DB Mirzaa GM Pagon RA Wallace SE Bean LJH, editors. GeneReviews(®). Seattle (WA): University of Washington, Seattle. Copyright © 1993–2022, University of Washington, Seattle. GeneReviews is a registered trademark of the University of Washington, Seattle. All rights reserved.; 1993.

[66] Finsterer J. Leigh And Leigh-like syndrome in children and adults. Pediatr Neurol (2008) 39(4):223–35. doi: 10.1016/j.pediatrneurol.2008.07.013 18805359

[67] Carelli V Sadun AA. Optic neuropathy in lhon and Leigh syndrome. Ophthalmology (2001) 108(7):1172–3. doi: 10.1016/S0161-6420(01)00618-2 11425664

[68] Stramkauskaitė A Povilaitytė I Glebauskienė B Liutkevičienė R. Clinical overview of leber hereditary optic neuropathy. Acta Med Litu (2022) 29(1):9–18. doi: 10.15388/Amed.2022.29.1.19 36061944 PMC9428633

[69] Stelmack JA Tang XC Reda DJ Rinne S Mancil RM Massof RW. Outcomes of the veterans affairs low vision intervention trial (LOVIT). Arch Ophthalmol (2008) 126(5):608–17. doi: 10.1001/archopht.126.5.608 18474769

[70] Barboni P Savini G Valentino ML La Morgia C Bellusci C De Negri AM. Leber's hereditary optic neuropathy with childhood onset. Invest Ophthalmol Vis Sci (2006) 47(12):5303–9. doi: 10.1167/iovs.06-0520 17122117

[71] Barboni P Savini G Valentino ML Montagna P Cortelli P De Negri AM. Retinal nerve fiber layer evaluation by optical coherence tomography in leber's hereditary optic neuropathy. Ophthalmology (2005) 112(1):120–6. doi: 10.1016/j.ophtha.2004.06.034 15629831

[72] Ramos Cdo V Bellusci C Savini G Carbonelli M Berezovsky A Tamaki C. Association of optic disc size with development and prognosis of leber's hereditary optic neuropathy. Invest Ophthalmol Vis Sci (2009) 50(4):1666–74. doi: 10.1167/iovs.08-2695 19098324

[73] Pfeffer G Horvath R Klopstock T Mootha VK Suomalainen A Koene S. New treatments for mitochondrial disease-no time to drop our standards. Nat Rev Neurol (2013) 9(8):474–81. doi: 10.1038/nrneurol.2013.129 PMC4967498 23817350

[74] Yu-Wai-Man P Soiferman D Moore DG Burté F Saada A. Evaluating the therapeutic potential of idebenone and related quinone analogues in leber hereditary optic neuropathy. Mitochondrion (2017) 36:36–42. doi: 10.1016/j.mito.2017.01.004 28093355 PMC5644719

[75] Meyerson C Van Stavern G McClelland C. Leber hereditary optic neuropathy: Current perspectives. Clin Ophthalmol (2015) 9:1165–76. doi: 10.2147/OPTH.S62021 PMC4492634 26170609

[76] Carelli V La Morgia C Valentino ML Rizzo G Carbonelli M De Negri AM. Idebenone treatment in leber's hereditary optic neuropathy. Brain (2011) 134(Pt 9):e188. doi: 10.1093/brain/awr180 21810891

[77] Klopstock T Metz G Yu-Wai-Man P Büchner B Gallenmüller C Bailie M. Persistence of the treatment effect of idebenone in leber's hereditary optic neuropathy. Brain (2013) 136(Pt 2):e230. doi: 10.1093/brain/aws279 23388409 PMC3572931

[78] Klopstock T Yu-Wai-Man P Dimitriadis K Rouleau J Heck S Bailie M. A randomized placebo-controlled trial of idebenone in leber's hereditary optic neuropathy. Brain (2011) 134(Pt 9):2677–86. doi: 10.1093/brain/awr170 PMC3170530 21788663

[79] Carelli V Carbonelli M de Coo IF Kawasaki A Klopstock T Lagrèze WA. International consensus statement on the clinical and therapeutic management of leber hereditary optic neuropathy. J Neuroophthalmol (2017) 37(4):371–81. doi: 10.1097/WNO.0000000000000570 28991104

[80] Ganti AK Chu ER Karanjia R Tran J Belfort R Jr Moraes M. EPI-743 may improve visual acuity in LHON: Data from a Brazilian cohort. Invest Ophthalmol Visual Sci (2014) 55(13):6201–.

[81] Zibold J von Livonius B Kolarova H Rudolph G Priglinger CS Klopstock T. Vitamin B12 in leber hereditary optic neuropathy mutation carriers: a prospective cohort study. Orphanet J Rare Dis (2022) 17(1):310. doi: 10.1186/s13023-022-02453-z 35945620 PMC9361590

[82] Newman NJ. Treatment of hereditary optic neuropathies. Nat Rev Neurol (2012) 8(10):545–56. doi: 10.1038/nrneurol.2012.167 22945544

[83] Saylor M McLoon LK Harrison AR Lee MS. Experimental and clinical evidence for brimonidine as an optic nerve and retinal neuroprotective agent: an evidence-based review. Arch Ophthalmol (2009) 127(4):402–6. doi: 10.1001/archophthalmol.2009.9 19365015

[84] Newman NJ Biousse V David R Bhatti MT Hamilton SR Farris BK. Prophylaxis for second eye involvement in leber hereditary optic neuropathy: an open-labeled, nonrandomized multicenter trial of topical brimonidine purite. Am J Ophthalmol (2005) 140(3):407–15. doi: 10.1016/j.ajo.2005.03.058 16083844

[85] Wan X Pei H Zhao MJ Yang S Hu WK He H. Efficacy and safety of rAAV2-ND4 treatment for leber's hereditary optic neuropathy. Sci Rep (2016) 6:21587. doi: 10.1038/srep21587 26892229 PMC4759604

[86] Liu HL Yuan JJ Zhang Y Tian Z Li X Wang D. Factors associated with rapid improvement in visual acuity in patients with leber's hereditary optic neuropathy after gene therapy. Acta Ophthalmol (2020) 98(6):e730–e3. doi: 10.1111/aos.14379 32096343

[87] Li X Tian Z Chen Z Li B Zhang Y. Efficacy evaluation of intravitreal injection of rAAV2-ND4 gene for leber hereditary optic neuropathy. Chin J Exp Ophthalmol (2021), 724–8.

[88] Zhang Y Tian Z Yuan J Liu C Liu HL Ma SQ. The progress of gene therapy for leber's optic hereditary neuropathy. Curr Gene Ther (2017) 17(4):320–6. doi: 10.2174/1566523218666171129204926 PMC5902861 29189152

[89] Newman NJ Yu-Wai-Man P Carelli V Moster ML Biousse V Vignal-Clermont C. Efficacy and safety of intravitreal gene therapy for leber hereditary optic neuropathy treated within 6 months of disease onset. Ophthalmology (2021) 128(5):649–60. doi: 10.1016/j.ophtha.2020.12.012 33451738

[90] Newman N Yu-Wai-Man P Carelli V Subramanian P Moster M Wang A-G. The phase III REFLECT trial: Efficacy and safety of bilateral gene therapy for leber hereditary optic neuropathy (LHON) (P17-12.002). Neurology (2022) 98(18 Supplement):928.

[91] Yu-Wai-Man P Newman NJ Carelli V Moster ML Biousse V Sadun AA. Bilateral visual improvement with unilateral gene therapy injection for leber hereditary optic neuropathy. Sci Transl Med (2020) 12(573). doi: 10.1126/scitranslmed.aaz7423 33298565

[92] Biousse V Brown MD Newman NJ Allen JC Rosenfeld J Meola G. *De novo* 14484 mitochondrial DNA mutation in monozygotic twins discordant for leber's hereditary optic neuropathy. Neurology (1997) 49(4):1136–8. doi: 10.1212/WNL.49.4.1136 9339703

